# Implementing the 2020–2025 Dietary Guidelines for Americans: Recommendations for a Path Forward

**DOI:** 10.1093/cdn/nzab136

**Published:** 2021-12-09

**Authors:** Lisa M Sanders, Jonathan C Allen, Jeanne Blankenship, Eric A Decker, Mary Christ-Erwin, Eric J Hentges, Julie M Jones, Farida Y Mohamedshah, Sarah D Ohlhorst, John Ruff, Jill Wegner

**Affiliations:** Cornerstone Nutrition, LLC, Battle Creek, MI, USA; Department of Food, Bioprocessing and Nutrition Sciences, North Carolina State University, Raleigh, NC, USA; Academy of Nutrition and Dietetics, Chicago, IL, USA; Department of Food Science, University of Massachusetts, Amherst, MA, USA; MCE Food & Agriculture Consulting, Annapolis, MD, USA; Retired, formerly with International Life Sciences Institute, North America, VA, USA; Department of Nutrition and Exercise Science, St. Catherine University, St. Paul, MN, USA; Institute of Food Technologists, Chicago, IL, USA; American Society for Nutrition, Rockville, MD, USA; Institute of Food Technologists, Chicago, IL, USA; Nestle, Fremont, MI, USA

**Keywords:** dietary guidelines, food science, nutrition communications, consumer trends, healthy eating pattern

## Abstract

The Dietary Guidelines for Americans (DGA) provide science-based recommendations for healthy dietary patterns to promote health and reduce risk of chronic diseases. Yet, since their inception in 1980 and updates every 5 y, Americans fall short of meeting dietary recommendations and diet-related chronic diseases continue to be a public health concern. In May of 2021, the Institute of Food Technologists and the Department of Food Science at the University of Massachusetts, Amherst, convened a diverse group of thought leaders in health, nutrition, and food science to identify opportunities and approaches to improve consumer adoption of the DGA recommendations. The invited leaders collaborated in roundtable discussions to develop recommendations and strategies to promote adoption of the DGA recommendations after hearing sessions on the latest consumer trends, advances in food science and technology, and effective communications approaches. Participants agreed that changes in consumer behaviors and heightened interest in health due to the novel coronavirus pandemic have created an opportune time to engage consumers about healthy eating. Communications must be simple, tailored to the consumer, and delivered by influencer(s)/spokesperson(s) who are credible sources and share personal values. Innovations in food science and technology have enabled improvements in the safety, health, acceptability, affordability, and availability of foods, but opportunities to provide more options to enhance consumption of desired food groups, such as fruits, vegetables, and whole grains, remain. Moving Americans toward healthier dietary patterns aligned with DGA recommendations will require collaborations within the food sector and beyond to achieve broad-scale amplification and investment.

## Teaser

Thought leaders in health, nutrition, and food science identified opportunities to improve consumer adoption of the *Dietary Guidelines for Americans 2020–2025* through better communication, collaboration, and innovation.

## Introduction

Since 1980, the Dietary Guidelines for Americans (DGA) have provided science-based guidance for healthy eating to meet nutrient needs and reduce risk of chronic diseases (1). The DGA are developed for health professionals and policymakers to enable the development and implementation of communications, programs, and policies to promote the health of the general population. For example, the recommendations within the DGA inform the dietary planning of federal nutrition programs, such as the National School Lunch Program and the Supplemental Nutrition Assistance Program. Additionally, the USDA's MyPlate, a consumer communication tool promoting healthy dietary patterns, is built upon the recommendations of the DGA (2).

Many DGA recommendations have remained relatively consistent and continue to build on previous editions with advances in food and nutrition science [e.g., changes in cholesterol intake recommendations; focus on dietary pattern(s) vs. nutrients and recommendations based on life stages] (1). Yet, consumer adoption of the DGA recommendations has been minimal, resulting in limited improvement in diet quality of Americans. Diet quality can be measured using the Healthy Eating Index, which provides a score from 0 to 100 based on alignment of the diet with dietary guidelines, such as limiting added sugars and saturated fats and consuming adequate fruits, vegetables, whole grains, and protein foods. From 2005 to 2016, the average Healthy Eating Index score of the US population increased from 56 to 59, falling far short of 100, the score indicating full adherence to the recommendations (1). Furthermore, diet-related chronic diseases, such as obesity, type 2 diabetes, and cardiovascular disease, continue to be major public health concerns, impacting 60% of US adults, and costing the US health care system billions of dollars each year (3).

Many reasons have been proposed for low adoption of the DGA recommendations by consumers, including inadequate knowledge, understanding, and motivation; economic and time barriers; and poor access to healthy foods. However, the recent coronavirus disease 2019 (COVID-19) pandemic has heightened consumer awareness about health and the importance of a healthy diet, particularly as individuals with diet-related chronic diseases are at greater risk of severe illness if infected with severe acute respiratory syndrome coronavirus 2 (SARS-CoV-2) (4). Thus, it is an opportune time to encourage and enable consumers to achieve healthy dietary patterns as outlined in the DGA and promote better health for the population. This paper summarizes the outcomes and recommendations from a 2-d meeting and panel and roundtable discussions on identifying approaches and strategies to improve implementation of the DGA recommendations.

## “Time to Kick Start Healthy Eating”—Convening the Experts

To improve implementation and adoption of the DGA recommendations, a better understanding of consumer needs, attitudes, and motivations that influence purchasing and consumption behavior is needed. Further, the identification and adoption of effective approaches to communicate and build trust are critical. It is also essential to examine new food science and technology opportunities to strive toward a safer and healthier food supply, while also meeting consumer needs and preferences.

In May of 2021, the Institute of Food Technologists^®^, in collaboration with the Department of Food Science at the University of Massachusetts at Amherst, organized a virtual meeting entitled “Time to Kick Start Healthy Eating,” with the objective to discuss opportunities and strategies for improved implementation and greater consumer adoption of the DGA recommendations. The meeting was funded by the USDA National Institute of Food and Agriculture (USDA/NIFA), grant number 2020–68015-31189, and included a multidisciplinary group of participants, such as policymakers; food scientists and technologists; dietitians; nutrition scientists; behavioral scientists; food, nutrition, and health communicators; and consumer advocates. The meeting agenda is provided in the **Supplemental Material**and the list of participants is in **Appendix A**.

Presentations and panel discussion on the 4th and 5th of May (open to public) and the roundtable discussion (35 invited participants) on May 11th focused on 3 themes: *1*) consumer trends, attitudes, and motivations towards diet and health; *2*) the role of science and technology innovations to help with improving the adoption of healthy dietary pattern(s), and *3*) effective communication and building trust. The focus of the meeting was to discuss opportunities to improve consumer adoption of the DGA recommendations. Discussions pertaining to the accuracy of the recommendations or whether they are optimal were beyond the scope of this meeting. Recordings of the public meetings can be found at https://www6.ift.org/Ecommerce/Meetings/MeetingDetail?productId=54886461.

The presentations focused on the translation of the DGA recommendations into communication tools, such as MyPlate; consumer health trends before and during the COVID-19 pandemic; the role of food science and technology innovations in enabling a safe, healthy, desirable, affordable, acceptable, and accessible food supply that meets consumer preferences; and strategies to effectively communicate and build consumer trust. The presentations and discussions emphasized principles that would apply to all recommendations within the DGA. However, in the food science and technology presentations, a few examples related to innovations to promote healthy dietary patterns, such as increasing intake of fruits, vegetables, whole grains, lean proteins, and seafood, and reducing intake of saturated fat, were presented.

These sessions served as a basis for subsequent panel and roundtable discussions that included virtual breakout sessions, to identify implementation strategies to increase consumers' ability to adopt and adhere to the DGA recommendations. All roundtable participants were invited to attend the presentations on 3–4 May. Participants who were unable to attend were provided recordings of the sessions prior to the roundtable discussions. Each participant attended 2 roundtable sessions on topics of their interest (consumer trends, role of science and technology, communications to build trust). The roundtable sessions were intentionally designed to be small (≤10 people) for candid discussion and provided all participants the opportunity to speak and offer diverse/opposing perspectives based on their professional experience and expertise.

## “Time to Kick Start Healthy Eating”—Presentations and Panel and Roundtable Discussions

### Consumer insights and how the pandemic changed consumer perceptions of health

#### Presentations

The meeting began with Stephenie Fu, Senior Policy Advisor at the USDA Center for Nutrition Policy and Promotion, presenting background on the key concepts of the 2020–2025 DGA, including healthy dietary patterns, healthy eating by life stage (with new recommendations for birth to age 2), and nutrient density. These concepts and a potential messaging framework were reviewed in focus groups of individuals from different life stages (e.g., parents of infants/toddlers and pregnant/lactating women), ages, socioeconomic status, and race/ethnicity to understand how to best communicate with consumers through USDA's MyPlate. The outcomes of the focus groups indicated that consumers had a positive perception about the concept of healthy dietary patterns, understood that healthy eating is important at every age, and recognized that these patterns can change based on life stages or when confronted with a health crisis, such as a diagnosis of a diet-related chronic condition. However, the concept of nutrient density was more difficult for consumers to understand, and the language used in the DGA for nutrition and health professionals to help consumers “make every bite count” was perceived as perhaps “extreme,” particularly for parents of young children.

The “Start Simple” framework for communicating to consumers about how to eat healthier was received very positively among consumer research participants. The core message states, “When it comes to changing your approach to healthy eating, start simple! Small changes matter and they make it easier for you to start today.” Consumers found the “Start Simple” approach encouraging, set the stage for success, and allowed for personalization and autonomy to choose from many options. Consumers found it easy to identify small steps to take toward eating healthier, such as introducing more vegetables into meals and reducing portion size. As a result, several resources, including quizzes, apps, and meal plans, were incorporated in MyPlate to enable consumers to set goals and get personalized recommendations on how to make simple dietary changes that add up over time.

**FIGURE 1:**
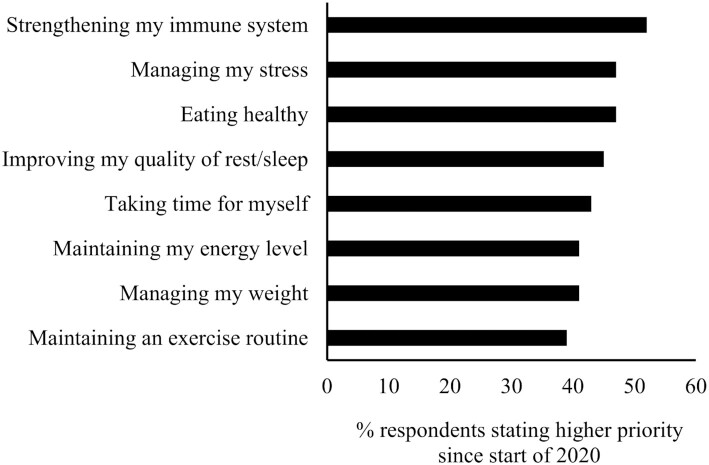
Increases in consumer priorities during 2020. Data from Lightspeed/Mintel (5) and based on 2000 internet users age ≥18 y. Bars represent the percentage of consumers ranking each outcome a higher priority since the start of January 2020.

**FIGURE 2:**
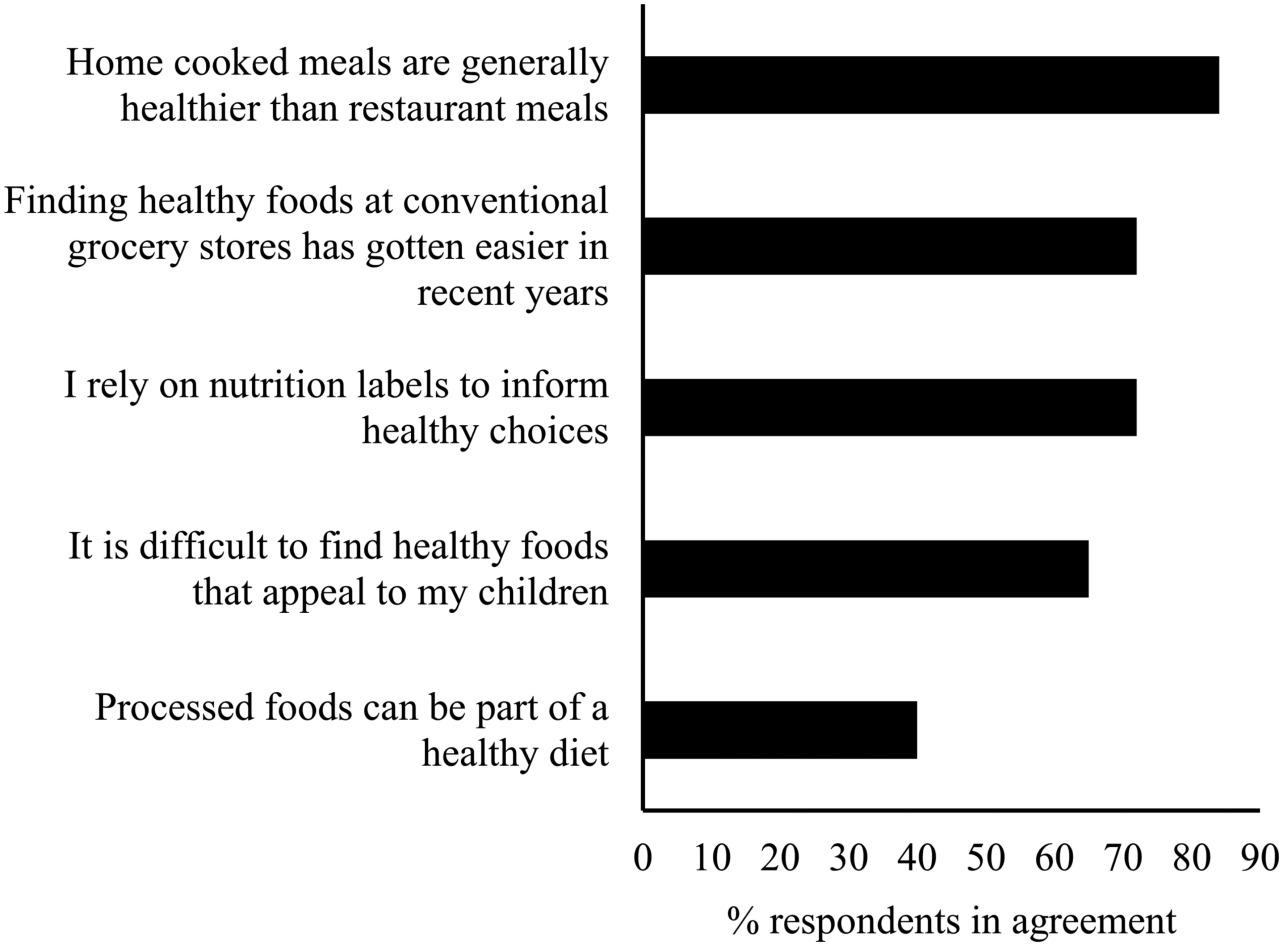
Consumer attitudes about healthy eating. Data from Lightspeed/Mintel (6) and based on 2000 internet users age ≥18 y. Bars represent the percentage of consumers agreeing with each statement.

Lynn Dornblaser, Director of Innovation and Insight at Mintel, discussed changes in consumer perspectives on health and healthy eating due to the COVID-19 pandemic. The data were obtained from consumer surveys of approximately 1800 to 2000 US adults, representative of the population. Health has become a greater priority for many since the beginning of the pandemic. Furthermore, consumers view health as not just physical but also emotional well-being. According to Mintel research presented at the meeting, the greatest health priorities in 2020 were “strengthening my immune system,” “managing my stress,” “eating healthy,” and “improving the quality of my rest/sleep” (**Figure 1**) (5). In fact, 47% of consumers reported regularly including “immunity-boosting” foods in their diet, which is up from 27% prior to 2020. Consumers also cooked at home more after the onset of the pandemic and 84% view home cooking as healthier than restaurant meals (**Figure 2**) (6). Home cooking includes cooking from scratch as well as incorporating convenient meal components available in stores. The prevalence of snacking has grown, with over half of consumers claiming to snack between meals regularly since the onset of the pandemic, but many do not expect to continue snacking as much in 2021 (7). The type of foods consumed as snacks was not evaluated, but almost half of consumers reported regularly eating indulgent foods since January of 2020. Like snacking, many expect to consume indulgent foods less often in 2021. The Generation Z (ages 18–24 y) and Millennials (ages 25–42 y) were more likely to report snacking and seeking indulgent foods. Additional trends included the growth of “plant-based” claims in the marketplace; however, only 18% of consumers identified plant-based as an attribute of a healthy food. Half or more of consumers identify “fresh” or “no/low sugar” as key attributes of healthy foods.

#### Panel and roundtable discussions

Based on these consumer insights and trends, panel and roundtable discussants identified several opportunities and strategies that should be considered for helping consumers choose a dietary pattern aligned with the DGA recommendations. These included the following:

- creating simple, consistent messaging;
- enhancing collaboration within the food and health sectors to expand strategic and targeted communication and training;
- leveraging the interest in home cooking and plant-based eating while addressing misperceptions; and
- offering more healthy food alternatives.

The panel and roundtable participants agreed that simple, consistent messaging is most effective for consumers and strongly supported the “Start Simple” approach. They agreed this messaging could be leveraged by all sectors, including policymakers, nutrition and health communicators, health professionals, the food industry, academics, advocacy organizations, and health-related nongovernmental organizations, to reinforce the messaging of MyPlate and the DGA. The participants indicated “Start Simple” strongly appeals to consumers because it takes aspirational goals, such as adherence to the totality of the DGA recommendations, and breaks them into small, easy-to-implement steps that can add up over time. Collaborations among stakeholders (identified above) to develop a unified and consistent approach and messaging may also help counter inconsistent and potentially confusing communications on healthy eating in traditional and social media.

Panelists and roundtable participants also identified opportunities for enhanced collaboration with other sectors, such as physicians, other health professionals, and the food industry, to expand the communications of the DGA. Most individuals do not see a dietitian but do see their primary care physician. However, physicians typically are not well versed in many aspects of nutrition, the recommendations of the DGA, and/or the scientific evidence supporting the recommendations. Thus, there is a need to raise physicians’ awareness and knowledge of nutrition science and healthy eating to make nutrition a key focus for health promotion and disease prevention. To accomplish this objective, expanded nutrition science coursework must be incorporated into medical training and continuing medical education.

In terms of the food industry, it was suggested that increased collaboration with retailers could strengthen messages of the DGA and potentially improve consumer adoption of the recommendations. As noted by panelist Krystal Register, Director of Health and Well-being at FMI–The Food Industry Association, “retailers have become a destination in the community for health and wellbeing,” supplying basic needs during the pandemic, such as soap and sanitizer, but also providing pharmacies, urgent care clinics, and the SARS-CoV-2 vaccine. In fact, a report by the FMI on grocery shopper trends revealed that two-thirds of consumers considered their primary food store to be on their side to help their family stay healthy (8).

The shift to online shopping, which increased due to the pandemic, will also likely continue as consumers find it easy and convenient. This trend creates another platform to encourage adherence to the DGA recommendations. The 2021 Food and Health Survey by the International Food and Information Council found that 42% of people purchased groceries online at least once a month, which is up from 33% in 2020 and 27% in 2019 (9). However, online shopping made it difficult for consumers to compare the nutrition profile of foods in absence of similar food options placed side-by-side on the store shelf. This presents an opportunity for industry and retailers to collaborate and improve online platforms to help direct consumers to healthy food products when selecting and placing the order. Further, through online platforms, retailers could provide tips and guidance on combining foods from various food groups into healthy meal patterns, thereby removing the emphasis on individual foods and focusing on the overall diet quality, per the DGA recommendations.

An increase in home cooking due to the pandemic was identified as an opportunity to address consumer perceptions about meals prepared (cooked) at home and offer more alternatives to make healthy meals at home. While many consumers believe home-cooked meals are healthier than restaurant meals, it really depends on the types of foods and ingredients used and how the foods are prepared at home or at a restaurant. Panelist Gareth Dutton, Professor, Diabetes Prevention and Control at the University of Alabama, Birmingham, noted that making healthy dietary choices in the home is based on availability, visibility, and accessibility. Healthy choices must be available in the home, they must be visible when looking into the pantry, and in some cases, they will need to be prepared (accessible) before they can be consumed. His perspectives about availability and accessibility at home go beyond the typical discussion around availability and accessibility in the store or supermarket to include knowledge of how to prepare foods (e.g., how to cook a squash). Availability, visibility, and accessibility at home are often the main challenges in adopting healthy eating pattern(s).

Participants agreed that enhanced communications on meal preparation and developing cooking skills could build on the momentum of home cooking to encourage consumption of healthy food products. Another related discussion was about destigmatizing certain food categories, such as canned and frozen foods, that are convenient, practical, affordable, and can be easily incorporated into a healthy dietary pattern but are often perceived as unhealthy or inferior because they are processed. This is particularly concerning as most consumers view “fresh” as the key attribute of healthy food and may be unaware that frozen or canned versions, especially those without added sugars and/or sodium, are healthy options as well. In fact, the DGA state that “all forms of foods, including fresh, frozen, canned and 100% juices, in nutrient dense forms, can be included in healthy dietary patterns” (1). Thus, there is an opportunity to build on the momentum of the home cooking trend by encouraging healthy alternatives for at-home meal prep. Additionally, continued innovation and reformulation by the food industry can provide more healthy and convenient options for at-home meal prep.

An opportunity to connect healthy eating to consumer values was identified as a critical motivator for consumers. For example, spending time with family is an important value for consumers, and cooking and eating together at home can reinforce this value. Research also suggests that all family members are more likely to consume fruits and vegetables when eating at home (10, 11), which aligns with DGA recommendations. Nutrition and health communicators and influencers, government, retailers, and industry could also emphasize the connection between these consumer values and the DGA recommendations.

Finally, the plant-based movement presents an opportunity to encourage fruit, vegetable, and whole-grain consumption. However, participants indicated that consumers may be unaware that plant-based is not synonymous with vegetarian, vegan, or elimination of meat and animal products, nor does it imply a food is healthy or less processed. Many foods that are plant-based, such as cookies or candy, are not nutrient-dense, whereas foods such as oatmeal, 100% orange juice, or whole-grain crackers can be healthy options, but many consumers may not think of these food products as plant-based. Helping consumers better understand the various plant-based options available and give suggestions for fitting them into healthy and culturally and ethnically appropriate dietary patterns may help them improve their diets.

### Role of food science and technology innovations in enabling a healthy food supply

#### Presentations

How science and technology innovations can enable a safe, healthy, affordable, desirable, and accessible food supply was the subject of presentations from food science researchers and food industry scientists. They focused on how such innovations can meet consumer needs, while at the same time help consumers follow the DGA recommendations. The presentation by Noel Anderson, Managing Partner at Mosaic Food Advisors and President of the Institute of Food Technologists, highlighted the application of food science, dating back to the prehistoric age with techniques such as salting, roasting, and sun drying to preserve and safely consume foods. Until the 19th century, most technologies in food science were focused on improving the safety and preservation of food, such as canning and pasteurization. However, as advances in nutrition science identified the importance of specific nutrients and their inadequacy for many in the population, food scientists addressed this issue with technologies such as fortification.

Many technological advancements occurred in the 20th century and continue today with techniques such as spray drying, freeze drying, high-pressure processing, and the development of artificial sweeteners, colors, flavors, and flavor enhancers (12). In addition to safety, nutrition, and convenience, many innovations today are driven by consumer needs and personal preferences as consumers “vote with their wallet.” This creates challenges as consumer demands are not always aligned with the DGA recommendations. Yet, with these challenges come great opportunities for the food industry to evolve their criteria for success and provide food products that meet consumer preferences while helping consumers adhere to the DGA recommendations.

Megan Bame, Extension Associate at North Carolina State University Plants for Human Health Institute and North Carolina Food Innovation Lab, examined how advances in crop genetics, pre- and postharvest techniques, and packaging technologies have all increased the accessibility and affordability of fruits and vegetables for consumers. Despite the myriad health benefits associated with fruit and vegetable consumption, such as reduced risk of cardiovascular disease and obesity (13–15), their intake remains far below the recommendations. Consumers cite several barriers to increased consumption, such as high cost and spoilage, as well as a lack of knowledge for how to choose and prepare fresh fruits and vegetables. However, some of these barriers are easy to overcome. For example, multiple apps exist that educate consumers on how to choose fresh fruits and vegetables in the store and provide numerous recipes for preparing vegetables, but consumers may not know about these.

Another barrier is the misperception that fruits and vegetables are expensive. While some fruits and vegetables are reported to be more expensive on a per pound basis, data from the USDA suggest an adult could meet the DGA recommendations for fruits and vegetables (e.g., fresh, canned, frozen, dried, 100% juice) for around $2.10 to $2.60 per day (16). Thus, fruits and vegetables may not be as expensive as perceived by consumers.

Spoilage continues to be a barrier to increased consumption of fruits and vegetables. Food scientists have developed technological advances to improve shelf-life, such as the use of genomic technologies to improve the resiliency of fruit and vegetable plants to different soils and climate conditions while not sacrificing the taste attributes that consumers desire (17). Additionally, the use of LED lighting in indoor farming and during postharvest storage has improved the shelf-life of fruits and vegetables as well as possibly enhancing nutritional quality (18). While packaging technologies, such as UV radiation and packaging films, have also minimized spoilage of fruits and vegetables, there are many promising technologies under evaluation, such as pulsed light, cold gas plasma, and high hydrostatic pressure, that may lengthen shelf-life and address spoilage issues to reduce some barriers of fruit and vegetable consumption (19). These processes can also increase availability of more different types of fruits and vegetables at a lower cost since spoilage is reduced.

Kevin Miller, Principal Scientist at General Mills, Inc., provided an overview of how advances in grain processing have enabled the addition of increasingly greater proportions of whole grains to more food products to help increase whole-grain consumption (20). Whole grains require processing—removal of the indigestible outer seed coat or hull before they can be consumed. Further milling helps to remove naturally occurring contaminants in the outer layers, such as mycotoxins and heavy metals, to improve food safety. Processing through cooking can improve the bioavailability of nutrients. Extrusion and heat from cooking breaks down the antinutrient phytate, releasing bound minerals for digestion and absorption. Heat processing can also free antioxidant-like compounds, such as ferulic acid, that are bound to dietary fibers, improving the nutritional quality and bioavailability of micronutrients and phytonutrients in whole-grain foods.

The final presentation of the session by Eric Decker, Professor in the Department of Food Science at the University of Massachusetts, Amherst, discussed recent technological advances and opportunities for developing healthy and sustainable high-protein foods. While most Americans meet or exceed protein recommendations, this is primarily achieved with meat, poultry, and eggs. More than half of Americans do not meet DGA recommendations for seafood, nuts, seeds, or soy products, which are important sources of protein and can also be lower in saturated fat than some animal protein sources (1). New technologies have enabled the development and availability of more varied sources of protein that are safe, convenient, and affordable. For example, seafood is typically more expensive and spoils quickly. Advances in packaging, such as flexible pouches for tuna, minimize spoilage, lengthen shelf-life, and reduce cost. Additionally, the flexible packaging requires less heat processing compared to canning, resulting in lower impact on sensory attributes, such as taste. Advances in high-pressure processing technologies may further improve flavor and texture. Technologies to bring more plant-based proteins to the market have rapidly accelerated in recent years. Brands such as Beyond Meat^®^ and Impossible have introduced plant-based meat alternatives to the mainstream. However, since these foods are designed to mimic meat texture and flavor, they can still be high in sodium and saturated fat. Additionally, they remain cost-prohibitive for low-income families.

Dairy foods are also a source of high-quality protein. New processing technologies, such as ultra-filtration and centrifugation, could increase the quantity of calcium and protein in milk and decrease the lactose for those who are lactose intolerant or want less sugar. However, these new dairy products are typically more expensive than traditional dairy foods. Technologies need to become more affordable to increase the availability and accessibility of these products to consumers across all income levels.

Dr. Decker proposed that the greatest opportunities for food technology exist for nuts, seeds, and legumes (beans, peas, lentils). For example, innovations to slow rancidity of unsaturated fats in nuts and seeds could increase shelf-life and enhance consumption. There are very few new processing technologies for these foods, even though they are a desirable source of plant protein, fiber, and bioactive compounds that are low in saturated fat compared with some animal protein sources.

#### Panel and roundtable discussions

Challenges, opportunities, and strategies for food and technology innovations to improve the adoption and adherence to the DGA recommendations were discussed with a broad group of stakeholders in roundtable discussions. Some of the key challenges identified included the following:

-  reformulation of established brands;
-  balancing health, taste, and affordability; and
-  meeting nutrient goals within some categories of foods.

Experts within the food industry discussed that consumers are looking for healthier alternatives to their favorite snacks and beverages. In response to consumer expectations and dietary recommendations, many food companies are pursuing reformulation, such as reduction of added sugars in foods and beverages. However, some consumers have emotional attachments to certain familiar foods and will quickly complain or stop purchasing a food if they notice slight changes in taste or appearance. Therefore, many well-established food products are difficult to reformulate and still maintain consumer acceptance. Thus, food companies reformulate existing products, as feasible, to avoid rejection due to preconceived appearance and taste expectations and introduce new healthy food products. Likewise, restaurants and foodservice establishments bring in new items, such as popular foods from other cultures, rather than altering consumer favorites.

Balancing health, taste, and affordability has long been a challenge, particularly with fruits and vegetables, as taste and affordability are often barriers for many consumers. However, Dr. Decker noted that there are many opportunities for science and technology to play a role in improving the taste profile of fruits and vegetables. For example, crop breeding and genetics have historically focused on yield, hardiness, or pest resistance, yet the technology can also be used to select for better taste profiles, such as reduced bitterness or enhanced sweetness. Innovative technology can also enable addition of fruits, vegetables, and other food groups to food products. Marianne O'Shea, Vice President of Global Health and Nutrition Sciences at PepsiCo, noted that some food companies are moving in this direction as consumers are seeking food groups over specific nutrients, a trend to be taken advantage of as it aligns with the DGA recommendations. By offering more products that incorporate food groups to encourage, consumers can make choices that work for themselves and their families. Yet, to be successful in making nutritious diets practical and acceptable to consumers, there is also a need to meet consumer demands for taste, convenience, and price. Affordability is often a large hurdle as many healthy foods can be more expensive. However, technologies such as canning, freezing, and specialized packaging and processing are available that can increase the shelf-life and reduce cost of food products.

Participants also expressed concern that the terms “healthy” and “nutrient dense” are not well defined or have outdated definitions. For example, the term “healthy” for use on food labels is defined by the FDA using nutrient content. Since the DGA have transitioned to recommending food groups and healthy dietary pattern(s) over nutrients, a new FDA definition of healthy or communications to show how a food fits into a healthy dietary pattern may be more useful.

Jessica Gould, Registered Dietitian and Director of Nutrition at Littleton Public Schools in Colorado, noted a “disconnect between the intention and the implementation” of the DGA, particularly for foodservice operators who are required to meet nutrient goals, such as sodium, for alignment with the DGA recommendations. For example, sodium is required to make bread, sauces, soups, and cheese (to make it safe for consumption), so planning monthly menus that provide a variety of foods from multiple food groups, meet nutrient goals, and deliver good taste is challenging. However, she commented that many innovations to provide food products that meet the “Smart Snack” guidelines (21) have greatly helped foodservice operators, and food companies should consider providing these snacks to consumers outside of schools since they align with the DGA recommendations.

Participants also identified the need for increased transparency so consumers can better understand how food is processed. Many methods (e.g., boiling, and canning) used by food companies to manufacture foods are similar to techniques used in home kitchens, only at a larger scale. Additionally, some foods, such as whole grains, require processing to be edible. Improving consumer understanding about food processing could also help reduce the stigma associated with processed food and increase the use of these foods in meal patterns that adhere to the DGA recommendations. The renewed consumer interest in science and technology due to the COVID-19 pandemic also provides an opportunity to communicate the important role of food science and technology in making more food options available to help consumers follow the DGA recommendations.

### Building consumer trust and effective communication to advance adoption of DGA recommendations

#### Presentations

Effective communications and building trust were the topics of the final sessions. Rosemary McGillan, Chief Marketing and Communications Officer at the American Red Cross, focused on 3 areas: the role of enhanced scrutiny by media and consumers, challenges in the current environment, and effective communication approaches to promote the DGA recommendations to consumers. With many social media and traditional media platforms, there is increased scrutiny on organizations, companies, government, and nonprofits. Thus, establishing and maintaining trust with the consumer is critical. Seventy-five percent of global consumers trust restaurants, food and beverage companies, and home care or household goods companies to act in their best interest (22), creating an opportunity for effective communication about the DGA recommendations by these trusted entities. Yet, there are challenges in the current culture, where digital media makes it difficult to decipher paid advertising from actual news, leading to confusing messages. Additionally, the current culture is highly argumentative and skeptical of truth. The “faceless” nature of social media has provided a platform where individuals are more comfortable sharing opinions and making it much easier to create disputes. Despite these challenges, there are also opportunities and solutions to better engage with consumers.

Consumers’ underlying beliefs and values can impact receptivity to messaging, and it is important to test messages to understand what a consumer “hears” rather than only what the message says. Media is fragmented and highly personalized, and the platforms used to obtain information vary by age groups and generations. Thus, communications must be tailored for the target audience and distributed through platforms that they are most likely to use. Finally, it is important to know whom consumers trust and whom they do not trust. Traditionally, education, degrees, and/or affiliations established credibility, but this is no longer sufficient as more people seek influencers with whom they can identify and who share their personal values.

Roxi Beck, Consumer Engagement Director at The Center for Food Integrity, and Vice President of Look East Consulting, expanded on the importance of personal values as a driver of consumer trust, utilizing “The Trust Model^SM^” (23). The Trust Model demonstrates that consumer trust begins with the people of influence in their social circles. Within these “influential others” the *competence* (knowledge, credentials, etc.) of the individual(s) is critical for driving trust, as is the *confidence* a consumer places in the individual(s) because of shared values and identity. Further, in building trust, consumer *confidence* in the individual is 3 to 5 times more important than the *competence* of the individual. To communicate effectively and to build trust, she recommended 3 behaviors to stop and 3 behaviors to start. Behaviors to stop include trying to persuade, correct/educate, or win arguments. While correcting misinformation and educating may be a potential outcome, it should not be the primary reason for the communication or conversation. Behaviors to start building trust include *1*) listening without judgement; *2*) asking questions to invite dialogue, clarity, and understanding; and *3*) sharing who you are, your perspective, and why you care.

#### Panel and roundtable discussions

Opportunities and strategies for effective communications and trust building needed to promote consumer adoption and adherence to the DGA recommendations were addressed during the panel and roundtable discussions. The need for diverse communicators, messages, and platforms to reach the many different types of consumers was recognized in all discussions. Messages need to be simple, tailored to the target audience, and delivered by influencers/communicators who can relate to the audience, but are also seen as credible and trustworthy. For example, the most credible source for information on the new guidelines for birth to 2 y of age might be health professionals who are also parents with children at this age and can relate to the personal struggles experienced in feeding young children. It was also noted that health is not the only motivator for consumers and that messaging should also consider additional motivators, such as personal values, that could encourage adoption and adherence to the DGA recommendations.

Partnership and collaboration with a wide variety of influencers are needed to develop unique, simple, and consistent messages and approaches that resonate with the audience and are also aligned with the DGA recommendations. Suggested influencers include behavioral scientists, marketers, sociologists, anthropologists, retailers, health care professionals, school educators and administrators, foodservice operators, insurance companies, professional athletes, celebrities, social media personalities, religious leaders, chefs, health coaches, and parents. The collaboration of these stakeholders, and many others, is crucial in helping Americans adopt a healthy dietary pattern(s) to improve public health.

Some participants advocated for grass-roots approaches, such as working in communities or within schools to affect behavior change, while others suggested national spokespeople for healthy eating. Adapting messaging to diverse socioeconomic, cultural, and health needs was also discussed. Specifically, messages on how healthy dietary patterns can be adopted for those with limited resources are critical for improving adherence to the DGA recommendations. Since food patterns differ by culture and health status, communications should give guidance on adapting the DGA recommendations into diverse cultural food patterns or to meet dietary constraints.

While participants were excited by the many opportunities to improve communications, they recognized that such a monumental effort could not be accomplished through the limited communication and marketing budget of the USDA and would require public–private partnerships and investments. Nancy Glick, Director of Food and Nutrition Policy at the National Consumers League, suggested that now may be the opportune time to advocate for another White House conference on nutrition to bring together key stakeholders for buy-in and garner the attention needed to affect large-scale investment and change. Finally, it was noted that metrics for success need to be developed to measure if communications impact behavior. The development of a metric(s) to measure healthy eating is complex because it needs to account for multiple dietary and behavioral modifications, making it more difficult than metrics for simple changes, such as getting vaccinated or wearing a safety belt.

### Recommendations/approaches for a path forward

“Time to Kick Start Healthy Eating” brought together diverse thought leaders from multiple sectors to identify opportunities and strategies to advance the implementation and adoption of the DGA recommendations by consumers. Equipped with the latest consumer insights, food science and technology advances, and evidenced-based strategies and approaches for effective communications and building trust, the recommendations and approaches discussed most frequently are included in **Box 1**.

**BOX 1. Recommendations and approaches to promote adoption and adherence to the DGA^1^**

**Recommendation 1:** Leverage the positive changes affected by the COVID-19 pandemic, including a greater focus on health and the prevalence of home cooking:

- Emphasize health benefits and other motivating values, such as increased family time, gained through cooking at home.
- Demonstrate the ease of incorporating healthy foods and ingredients from multiple types of processing into home-cooked meals.
- Empower consumers with cooking skills and methods to prepare a variety of foods that can be a part of a healthy dietary pattern.

**Recommendation 2:** Continue to promote a safe, healthy, affordable, accessible, and consumer-acceptable food supply:

- Advance food science and technology to expand a healthy and safe food supply and drive toward making foods created with these technologies more affordable for all consumers.
- Leverage the current interest in science to debunk myths about food processing by demonstrating the similarity of techniques used to make foods at home and at scale in food industry, to show how food processing can contribute to the solution.
- Destigmatize food categories, such as canned and frozen, to expand consumers understanding of the many foods that can help them conveniently and affordably meet the DGA recommendations.

**Recommendation 3:** Broaden collaborations and partnerships within the food sector and beyond:

- Unify and amplify the messages of the DGA.
- Advocate for public–private partnerships and investment to start moving dietary patterns closer to the DGA recommendations.
- Require adequate nutrition and health promotion training of primary care physicians and other health care professionals and offer continuing education for practitioners.
- Partner with retailers to amplify messages in store and online to nudge consumers to healthier meal patterns.
- Better understand and learn from the challenges and successes of implementation of the DGA with large populations, such as in school meals and foodservice

**Recommendation 4:** Employ communication strategies to build trust:

- Move away from traditional communication approaches that focus on education to strategies that meet consumers where they are to affect small changes in diet that add up over time.
- Develop targeted, simple, consistent messages for different demographics, age, cultures, races, values, motivators, etc.
- Employ a diverse set of communication platforms commonly used by the targeted audience.
- Engage influencers/spokesperson(s) the target consumer will relate to and trust.

^1^COVID-19, coronavirus disease 2019; DGA, Dietary Guidelines for Americans.

## Conclusions

The Secretaries of the USDA and Department Health and Human Services (HHS) stated in the 2020–2025 DGA (1):

“It's more important than ever to make healthy eating a priority in the United States. With the release of the *Dietary Guidelines for Americans, 2020-2025*, we have an important call to action for you as health professionals and policymakers. We are asking you to help the public “make every bite count with the *Dietary Guidelines for Americans*.” Help people make food and beverage choices that are rich in nutrition—individual choices that can become a healthy routine over time, choices they can enjoy in good health for many years to come.”

The 2-d meeting and the roundtable discussions extended the USDA and HHS “call to action” beyond health professionals and policymakers, by engaging stakeholders from many sectors—government, industry, consumer groups, health care professionals, academia, communications, and others—to identify strategies and approaches to help improve the adoption and adherence of the DGA recommendations for better health outcomes.

The recommendations and approaches identified may seem ambitious, but as MyPlate encourages consumers to Start Simple, stakeholders must also “start simple,” collaborate, partner, build upon, and utilize the many strengths they collectively bring to help improve the adoption of the DGA recommendations by consumers. This monumental task cannot be accomplished by the government alone or even a handful of key stakeholders. All public and private entities invested in the health of Americans play a crucial role in promoting the DGA recommendations, encouraging and empowering consumers with tools and resources to make small positive shifts and to build on these over time to improve health of Americans and the nation. It is only through the concerted and unified efforts of many stakeholders that the ultimate goal can be achieved.

## Supplementary Material

nzab136_Supplemental_File

## Notes

The meeting was funded by the USDA's National Institute of Food and Agriculture, grant number 2020-68015-31189 (EAD obtained funding). The funds were used to support preparation of the manuscript, moderator honoraria, and public relations. Additionally, a portion of the funds were provided to the Institute of Food Technologists® to support meeting promotion, online registration, and logistics for a virtual meeting.

Author disclosures: MC-E is President and Owner of MCE Food & Agriculture Consulting and received an honorarium from the grant for moderating the meeting and panel and roundtable discussions. JMJ is a Scientific Advisor to USA Rice, Grain Foods Foundation, and the Quality Carbohydrate Coalition. FYM was employed by the Institute of Food Technologists at the time of submission of publication. Following submission she changed roles and is currently at the National Confectioner's Association. JR is an Investment Committee Member for Sathguru Catalyser Advisors Private Limited, the Asset Management Company of Innovation in Food & Agriculture Fund (IFA Fund) that invests in innovation-driven growth enterprises in the Food and Agriculture sectors, based in India. He is reimbursed for meeting fees and expenses related to attending committee meetings but has no investments in the fund. LMS is the owner of Cornerstone Nutrition, LLC, a consultancy that has received funding from Kellogg Company, PepsiCo, and The Coca-Cola Company. LMS received writing fees from the grant for development of this manuscript. JW is an employee of Nestle. The other authors report no conflicts of interest.

Participants named in the manuscript are aware and have confirmed the inclusion of their name in the manuscript.

Abbreviations used: COVID-19, coronavirus disease 2019; DGA, Dietary Guidelines for Americans; HHS, Department of Health and Human Services; SARS-CoV-2, severe acute respiratory syndrome coronavirus 2.

## APPENDIX A

**Planning Committee Members:** Jonathan Allen, Department of Food, Bioprocessing and Nutrition Sciences, North Carolina State University, Raleigh, NC; Jeanne Blankenship, Academy of Nutrition and Dietetics; Eric Decker, Department of Food Science, University of Massachusetts, Amherst; Mary Christ-Erwin, MCE Food & Agriculture Consulting; Eric Hentges, Retired, formerly with International Life Sciences Institute; Julie M. Jones, St. Catherine University; Farida Mohamedshah, Institute of Food Technologists; Sarah D Olhorst, American Society of Nutrition; John Ruff, Institute of Food Technologists; Jill Wegner, Nestle.

**Moderator:** Mary Christ-Erwin, MCE Food & Agriculture Consulting.

**Speakers:** Noel Anderson, Mosaic Food Advisors, President Institute of Food Technologists; Megan Bame, Plants for Human Health Institute, Food Innovation Lab, North Carolina State University; Roxi Beck, The Center for Food Integrity and Look East Consulting; Eric Decker, Department of Food Science, University of Massachusetts, Amherst; Lynn Dornblaser, Mintel; Stephenie Fu, Center for Nutrition Policy and Promotion, Food and Nutrition Service, US Department of Agriculture; Rosemary McGillan, American Red Cross; Kevin Miller, General Mills’ Bell Institute of Health and Nutrition.

**Panelists:** Gareth Dutton, School of Medicine/Division of Preventive Medicine, University of Alabama, Birmingham; Katherine Jacobs, Food Research & Action Center; Janet de Jesus, Office of Disease Prevention and Health Promotion, US Department of Health and Human Services; Marisa Moore, Marisa Moore Nutrition; Marianne O'Shea, PepsiCo; Krystal Register, FMI-The Food Industry Association.

**Roundtable facilitators and scribes:** Jonathan Allen, Department of Food, Bioprocessing and Nutrition Sciences, North Carolina State University, Raleigh, NC; Jeanne Blankenship, Academy of Nutrition and Dietetics; Mary Christ-Erwin, MCE Food & Agriculture Consulting; Julie M. Jones, St. Catherine University; Sarah D. Olhorst, American Society of Nutrition; Louise Pollock, Pollock Communications; John Ruff, Institute of Food Technologists; Lisa M Sanders, Cornerstone Nutrition, LLC.

**Roundtable participants:** Steve Abrams, Department of Pediatrics, The University of Texas at Austin, Dell Medical School; Anne Agler, Abbott Nutrition; Bill Aimutis, North Carolina Food Innovation Lab; Noel Anderson, Mosaic Food Advisors, President Institute of Food Technologists; Jamy Ard, Department of Epidemiology and Prevention and the Department of Medicine, Wake Forest University Baptist Medical Center; Roxi Beck, The Center for Food Integrity and Look East Consulting; Eric Decker, Department of Food Science, University of Massachusetts, Amherst; Janet de Jesus, Office of Disease Prevention and Health Promotion, US Department of Health and Human Services; Lynn Dornblaser, Mintel; Gareth Dutton, School of Medicine/Division of Preventive Medicine, University of Alabama, Birmingham; Stephenie Fu, Center for Nutrition Policy and Promotion, Food and Nutrition Service, US Department of Agriculture; Nancy Glick, National Consumers League; Stephanie K Goodwin, Danone North America; Jessica Gould, Littleton Public Schools, Colorado; Thomas Gremillion, Consumer Federation of America; Geri Henchy, Food Research & Action Center; Wendy Johnson, Nestle USA; Wendelyn Jones, Institute for the Advancement of Food and Nutrition Sciences; Susanne Kettler, Mondelez International; Richard D Mattes, Purdue University; Rosemary McGillan, American Red Cross; Robin McKinnon, Food and Drug Administration, Center for Food Safety and Applied Nutrition; Linda Novick O'Keefe, Common Threads; Marianne O'Shea, PepsiCo; Krystal Register, FMI–The Food Industry Association; Kristin Reimers, Conagra Brands; Kristin Herron Rubin, Mars, Inc.; Barbara O Schneeman, University of California at Davis (Emeritus); Jessi Silverman, Center for Science in the Public Interest; Jessica Smith, General Mills; Jasia Steinmetz, Society for Nutrition Education and Behavior; Cheryl D Toner, American Heart Association; Ashley Vargas, Eunice Kennedy Shriver National Institute of Child Health and Human Development's Pediatric Growth and Nutrition Branch; Allison Dostal Webster, International Food Information Council; Amanda Young, Kraft Heinz Company.
