# Supplementary material for: Implementing the 2020–2025 Dietary Guidelines for Americans: Recommendations for a Path Forward
Source: Curr Dev Nutr. 2021 Dec 9;5(12):nzab136. doi: 10.1093/cdn/nzab136 (PMC8662785; doi:10.1093/cdn/nzab136)
Supplement: nzab136_Supplemental_File [file nzab136_supplemental_file.pdf]

Implementing the 2020-2025 Dietary Guidelines for Americans: Recommendations for a Path Forward

Lisa M. Sanders, et al.

Online Supplemental Material

Meeting Agenda – Time to Kick Start Healthy Eating

Tuesday, May 4, 1 – 3:30 pm ET

1:00 pm Welcome: Mary Christ Erwin, President, MCE Food & Agriculture Consulting

1:15 pm Insights from the Dietary Guidelines for Americans and MyPlate

- Speaker: Stephenie Fu, USDA Center for Nutrition Policy and Promotion

1:30 pm How the pandemic has shaped consumers' notions of health

- Speaker: Lynn Dornblaser, Mintel International Group Ltd.

2:00 pm Panel discussion: Strategies to Kick Start Healthy Eating

- Gareth Dutton, School of Medicine/Division of Preventive Medicine, University of Alabama, Birmingham
- Katherine Jacobs, Food Research & Action Center
- Janet de Jesus, Office of Disease Prevention and Health Promotion, US Department of Health and Human Services
- Marisa Moore, Marisa Moore Nutrition
- Marianne O'Shea, PepsiCo
- Krystal Register, FMI-The Food Industry Association

3:15 pm Concluding Remarks and Wrap up: Mary Christ Erwin

Wednesday, May 5, 1 – 3:30 pm ET

1:00 pm Welcome and Recap of Day 1: Mary Christ Erwin, MCE Food & Agriculture Consulting

1:15 pm Enabling a Healthy, Desirable, and Accessible Food Supply

- Speaker: Noel Anderson, Mosaic Food Advisors, President Institute of Food Technologists

1:30 pm How Science and Technology Meets the Challenge

- Can Technical Advances Encourage Produce Consumption?
  - Speaker: Megan Bame, Plants for Human Health Institute, Food Innovation Lab, North Carolina State University
- Food Industry Journey to Improve Diet Quality Through Whole Grain
  - Speaker: Kevin Miller, General Mills
- New Opportunities for Healthy and Sustainable High Protein Foods

    - Speaker: Eric Decker, Department of Food Science at the University of Massachusetts, Amherst

2:30 pm Communicating and Building Trust

- It's about choices: Strategies for Effective Communication
    - Speaker: Rosemary McGillan, American Red Cross
- The Trust Factor
    - Speaker: Roxi Beck, Center for Food Integrity

3:15 pm Concluding Remarks and Wrap up: Mary Christ Erwin

<u>Tuesday, May 11, 1 – 3:15 pm ET</u>

1:00 pm Welcome – John Ruff, Institute of Food Technologists & Mary Christ-Erwin, MCE Food & Agriculture Consulting

1:15 pm – 2:30 pm Two Sessions of Roundtable Discussions – (≤ 10 participants/discussion)

- Topic 1: Consumer trends
- Topic 2: Opportunities and challenges: Role of science and technology innovations
- Topic 3: Communicating and building trust to improve adoption of the DGAs
- Facilitators: Jeanne Blankenship, Academy of Nutrition and Dietetics; Mary Christ-Erwin, MCE Food & Agriculture Consulting; Julie Miller Jones, St. Catherine University; John Ruff, Institute of Food Technologists

2:30 pm Break for facilitators to summarize roundtable discussions

2:50 pm Key outcomes from the breakout sessions presented by facilitators

3:10 pm Wrap up – John Ruff, Institute of Food Technologists
